# Isolated Cerebral Metastasis in Ovarian Cancer: A Report of a Rare Case

**DOI:** 10.7759/cureus.103905

**Published:** 2026-02-19

**Authors:** Rayen Cherif, Haykel Turki, Samir Aloulou, Ines Saguem, Hamdani Moez

**Affiliations:** 1 Medical Oncology, University Hospital of Gabes, Gabes, TUN; 2 Surgical Oncology, Institute of Salah Azaïz, Tunis, TUN; 3 Medical Oncology, University of Sfax, Sfax, TUN; 4 Pathology, University Hospital of Gabes, Gabes, TUN; 5 Pathology, Sadok Mokaddem Regional Hospital, Djerba, TUN

**Keywords:** brain metastasis, cns involvement, neurosurgical management, ovarian cancer, targeted therapy

## Abstract

Brain metastases in ovarian cancer are uncommon, yet they signify aggressive disease and are associated with limited survival. This case highlights the diagnostic challenges and therapeutic dilemmas in managing central nervous system (CNS) involvement in ovarian cancer, given the lack of standardized protocols. This report adheres to the Surgical Case Report (SCARE) 2025 checklist. A 58-year-old woman with stage IIIB high-grade serous ovarian carcinoma in systemic remission developed a solitary frontal lobe metastasis 18 months after initial diagnosis. The diagnosis was suspected on MRI and confirmed by histopathological analysis after gross total resection of the tumor. While surgical intervention, as in this case, can provide symptomatic relief and pathological confirmation, the prognosis for patients with CNS metastases from ovarian cancer remains poor. This underscores the need for two key approaches in high-risk patients (e.g., those with residual postoperative tumor or unfavorable molecular markers): first, enhanced surveillance, implying more frequent imaging and a lower threshold for initiating adjuvant therapy, and second, the exploration of novel CNS-penetrating therapies. This case illustrates that even with aggressive local treatment for a solitary brain metastasis, outcomes are often unfavorable. It emphasizes the importance of a high index of suspicion for neurological symptoms in ovarian cancer patients and highlights the urgent need for more effective treatment strategies.

## Introduction

Ovarian cancer remains the most lethal gynecologic malignancy, largely due to its frequent detection at an advanced stage, a consequence of its vague and nonspecific symptomatology. High-grade serous carcinoma (HGSC), the most common type of ovarian cancer, represents the predominant and most aggressive histologic subtype, commonly characterized by widespread peritoneal disease and distant metastatic spread. Cerebral metastases are an uncommon complication of ovarian cancer, reported in fewer than 2% of cases, and are linked to an especially unfavorable prognosis [1]. Without treatment, median survival is typically limited to 3-16 months [2]. However, patients with a single brain metastasis may survive longer than those with multiple lesions [3]. 

Although traditionally viewed as a rare terminal event, there is emerging discussion that the frequency of brain metastases in ovarian cancer patients could be increasing. This trend is possibly attributable to enhanced systemic therapies that extend overall survival but may not adequately protect against hematogenous dissemination to sites like the central nervous system (CNS) [4]. This evolving clinical scenario highlights a significant deficiency in conventional treatment protocols. The biological drivers of this specific metastatic pattern, including potential associations with molecular profiles such as BRCA1/2 mutation status, therapeutic-driven clonal evolution, or other factors, are an active area of investigation. Notably, emerging evidence suggests that BRCA-mutated ovarian cancers may have a higher propensity for CNS spread [5]. Additionally, the scarcity of such cases has resulted in a notable absence of robust clinical trials, leaving optimal management strategies unclear. Treatment decisions typically require a nuanced, multimodal approach combining neurosurgical intervention, radiation, and chemotherapy [6].

This work details the case of a 58-year-old female patient diagnosed with stage IIIB ovarian cancer, as per the classification of the International Federation of Gynecology and Obstetrics (FIGO) [7], who subsequently developed an intracranial metastasis following standard primary treatment involving neoadjuvant chemotherapy and interval cytoreductive surgery. Consequently, the objectives of this case analysis are twofold: firstly, to provide a comprehensive clinical account that augments the limited data available on this rare manifestation, with specific attention to its unique presentation timeline and histopathologic characteristics; and secondly, to evaluate the implemented treatment regimen within the context of the current, constrained evidence landscape. Through this examination, the report aims to underscore diagnostic difficulties, emphasize the importance of clinical vigilance, and highlight the integrated multidisciplinary planning essential for the care of such patients.

This report has been prepared in accordance with the Surgical Case Report (SCARE) 2025 revised checklist to ensure standardized and transparent case reporting [8].

## Case presentation

A 58-year-old woman was diagnosed with high-grade serous ovarian carcinoma (FIGO stage IIIB) with peritoneal carcinomatosis and hepatic metastasis in November 2023. BRCA germline testing was attempted but yielded inconclusive results due to technical limitations. She received six cycles of neoadjuvant carboplatin-paclitaxel, achieving a 49% pelvic tumor response and complete hepatic remission, followed by optimal cytoreduction (hysterectomy, bilateral salpingo-oophorectomy, omentectomy, and appendectomy). Histopathology confirmed residual HGSC. Four adjuvant chemotherapy cycles were completed.

**Figure 1:**
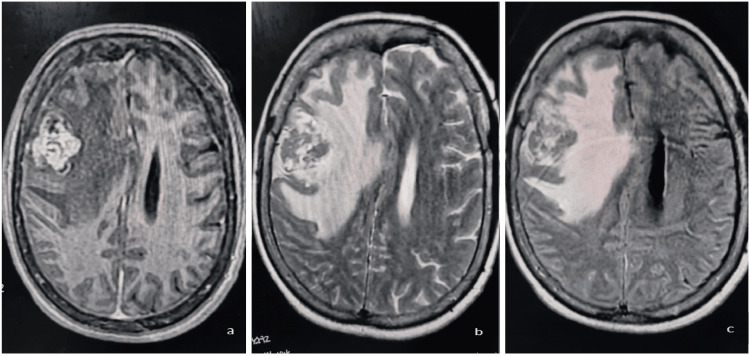
Brain MRI showing heterogeneous tumor with central necrosis, peritumoral edema, and leftward midline shift in right frontal lobe. (a) T1W sequence showing a hypointense lesion with heterogeneous internal areas. (b) T2W sequence demonstrating a heterogeneous hyperintense mass. (c) FLAIR sequence showing bright heterogeneous signal. T1W: T1-weighted; T2W: T2-weighted; FLAIR: fluid-attenuated inversion recovery

In May 2025, she presented with acute left-sided hemiparesis and blurred vision. Neurological examination revealed severe left hemiparesis (Medical Research Council (MRC) Scale for Muscle Strength grade 2/5 in the upper and lower limbs) [9], left-sided facial weakness, and evidence of bilateral papilledema on fundoscopy. Brain MRI revealed a 36-mm right frontal lobe mass with vasogenic edema, initially suggestive of high-grade glioma; however, systemic imaging confirmed peritoneal and nodal recurrence (Figure 1).

**Figure 2:**
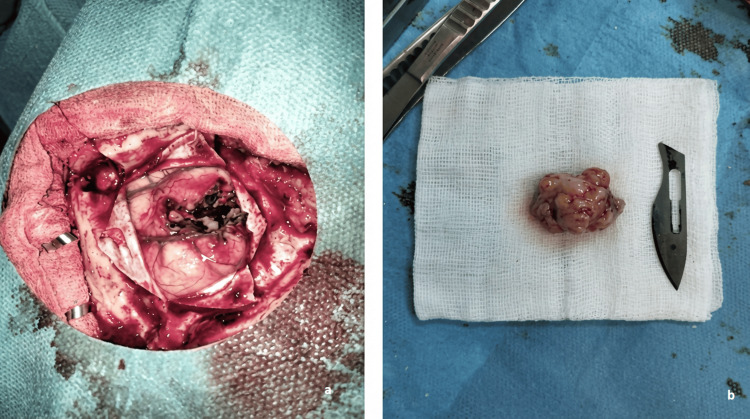
Surgical resection of right frontal metastasis. (a) Intraoperative view of tumor resection cavity. (b) Resected tumor specimen.

**Figure 3:**
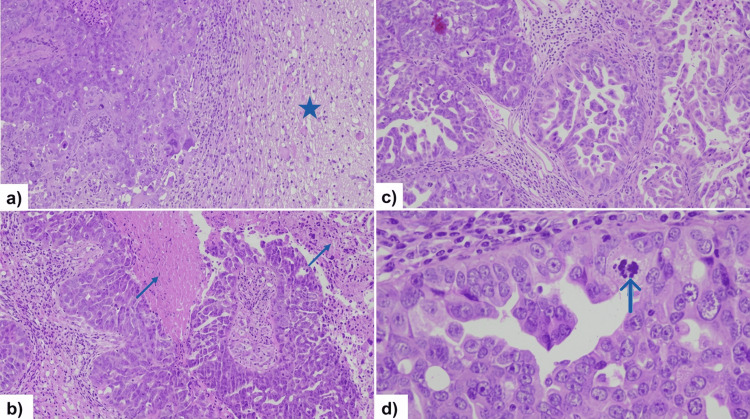
Brain metastasis exhibiting solid, papillary, and micropapillary patterns with nuclear atypia. (a,b) Cerebral infiltration (star) by carcinomatous proliferation organized in solid sheets and papillae with areas of necrosis (arrows) (HE x100). (c) Variable architectural features, including papillary and micropapillary patterns (HE x200). (d) Tumor cells with marked nuclear pleomorphism, prominent nucleoli, and mitotic activity (arrow) (HE x400). HE: hematoxylin and eosin

Given her oncological history, a metastatic lesion from her ovarian primary was suspected. The patient underwent neurosurgical resection given the diagnostic uncertainty, as the heterogeneous imaging appearance with central necrosis and extensive peritumoral edema mimicked a high-grade glioma, necessitating tissue diagnosis. Stereotactic radiosurgery (SRS) was not considered a viable option due to the need for definitive histopathological confirmation, the significant mass effect with leftward midline shift requiring urgent surgical decompression, and the large tumor size with symptomatic edema. Gross total resection was successfully achieved. Intraoperatively, the tumor demonstrated well-circumscribed margins with a distinct tumor-brain interface. Histopathological examination confirmed metastatic HGSC, consistent with her primary ovarian cancer (Figures 2-3). Immunohistochemistry revealed positivity for PAX8, WT1, and CK7, with negativity for CK20 and TTF-1, confirming the ovarian origin of the metastasis and excluding primary brain tumor or metastases from other sites.

Postoperative follow-up demonstrated significant clinical improvement, confirmed by objective measures. Neurological examination revealed complete resolution of the left hemiparesis, with motor strength improving from MRC grade 2/5 to 4/5 in the affected limbs. Fundoscopy showed complete resolution of the previously noted bilateral papilledema, indicating successful alleviation of intracranial pressure. The patient is currently receiving second-line chemotherapy with an association of doxorubicin and gemcitabine. BRCA mutation testing results were not available, precluding consideration of PARP inhibitor therapy. Additionally, bevacizumab was not available at our institution, and the patient lacked national health insurance coverage (Caisse Nationale de l'Assurance Maladie (CNAM)), which limited access to costly targeted therapies. Treatment selection was therefore based on the platinum-free interval, prior chemotherapy exposure, and institutional drug availability.

## Discussion

Brain metastases in ovarian cancer are uncommon but indicate aggressive disease biology [10,11]. The incidence is increasing, possibly due to improved systemic control leading to longer survival, allowing metastatic spread to the CNS [12]. However, in our patient, brain metastasis developed relatively early, approximately 18 months after initial diagnosis (November 2023 to May 2025), suggesting particularly aggressive disease biology rather than prolonged survival leading to CNS sanctuary site progression. The frontal lobe is a frequent site of metastasis, as seen in this case.

Diagnosis relies on imaging, with MRI being the gold standard, and histopathological confirmation. Differential diagnoses include primary brain tumors, particularly glial tumors, infections, or vascular lesions, but a history of malignancy should raise suspicion for metastasis.

Treatment options include surgical resection (for solitary, accessible lesions), SRS, whole-brain radiotherapy (WBRT), and systemic therapy. Management of solitary brain metastases requires multidisciplinary decision-making involving neurosurgery, medical oncology, radiation oncology, and neuroradiology. In our patient, a multidisciplinary tumor board discussion led to the decision for surgical resection given the solitary, accessible frontal lobe location, diagnostic uncertainty requiring tissue confirmation, and significant mass effect with neurological symptoms necessitating urgent decompression. Although CNS involvement in ovarian cancer is rare and optimal management remains unclear, surgical resection provided both symptomatic relief and definitive diagnosis.

Prognosis is generally poor, with median survival ranging from three to 16 months after diagnosis of brain metastasis [2]. Recent studies suggest that BRCA mutations may increase the risk of brain metastases in ovarian cancer [13,14]. While this patient’s mutational status was not conclusive, future testing could guide targeted therapies (e.g., PARP inhibitors) to improve outcomes. Factors influencing survival include performance status, extent of extracranial disease, and treatment modality. The prognosis for ovarian cancer patients with brain metastases remains dismal, emphasizing the need for early detection and multidisciplinary care [15]. Further research is warranted to establish standardized treatment protocols, including the role of immunotherapy and blood-brain barrier-penetrating agents.

## Conclusions

This case demonstrates several key clinical lessons directly supported by our patient's presentation and course. First, brain metastasis should be considered in ovarian cancer patients presenting with acute neurological symptoms, even relatively early in the disease course (18 months post-diagnosis in this case). Second, when imaging features mimic primary brain tumors - as occurred here with heterogeneous signal, central necrosis, and extensive edema - surgical resection serves the dual purpose of providing definitive tissue diagnosis and immediate symptom relief from mass effect. Third, multidisciplinary tumor board discussion involving neurosurgery, medical oncology, and radiation oncology was essential in this case and should be standard practice for all patients with brain metastases. This case also illustrates significant real-world barriers to optimal care that directly influenced treatment decisions: BRCA mutation testing results were not available at the time of treatment initiation, bevacizumab was not on our institutional formulary, and the patient lacked national health insurance coverage (CNAM), which restricted access to targeted therapies. These constraints necessitated the use of doxorubicin and gemcitabine rather than guideline-preferred options. This experience underscores the importance of performing BRCA/homologous recombination deficiency (HRD) testing at initial ovarian cancer diagnosis to inform future treatment decisions, particularly as PARP inhibitors and other targeted therapies become increasingly important in recurrent disease management.

Given the rarity of brain metastases in ovarian cancer, disease-specific treatment protocols are lacking. While robust general guidelines exist for managing brain metastases (e.g., American Society of Clinical Oncology (ASCO)/Society for Neuro-Oncology (SNO) and the National Comprehensive Cancer Network (NCCN)), critical questions remain unanswered for this specific population, including the optimal systemic therapy approach, the role of blood-brain barrier-penetrating agents, and the sequencing of neurosurgical intervention, radiation, and systemic therapies. Further research is needed to establish evidence-based, ovarian cancer-specific management protocols and to address healthcare system barriers that create disparities in access to molecular diagnostics and targeted therapies.
